# From hype to hope: reanimating phage therapy through evidence-based multidisciplinarity

**DOI:** 10.1038/s41467-026-72590-7

**Published:** 2026-05-07

**Authors:** Aleksandra Petrović-Fabijan, Stephen T. Abedon

**Affiliations:** 1 Centre for Infectious Diseases and Microbiology, Westmead Institute for Medical Research, Sydney, NSW Australia; 2 School of Medicine, Sydney Medical School, University of Sydney, Sydney, NSW Australia; 3 Sydney Infectious Diseases Institute (Sydney ID), University of Sydney, Sydney, NSW Australia; 4 Department of Microbiology, The Ohio State University, Mansfield, OH USA

**Keywords:** Bacteriophages, Phage biology, Bacteriology, Translational research, Applied microbiology

## Abstract

Phage therapy must move beyond anecdote to clinical reproducibility. Despite compelling cases of bacteriophages curing otherwise untreatable infections, inconsistent outcomes and biological complexity continue to limit clinical adoption. Here we identify three foundational pillars–drug delivery, treatment effectiveness, and managing bacterial resistance that must be addressed through interdisciplinary collaboration to transform phage therapy from a niche intervention to a powerful weapon against antimicrobial resistance. The time for rigorous, translational phage science is now.

## Abstract (web summary)

Petrovic Fabijan and Abedon propose three foundational pillars — pharmacokinetics, pharmacodynamics, and fighting evolution — as an evidence-based framework to advance phage therapy from compelling anecdotes to reproducible clinical outcomes.

## Introduction

The global threat of multidrug resistance is escalating, pushing conventional antibiotics to their limits^1,2^. Even bacteria deemed antibiotic sensitive can cause chronic infections that defy cure, especially within an aging population. Procedures once considered routine—like artificial joint replacements—now carry heightened risks, particularly for immunocompromised individuals, while those with cystic fibrosis or organ transplants face persistent, life-threatening infections^3,4^. With resistance accelerating beyond the pace of antibiotic development, countless patients are left without effective options. The need for viable, alternative antibacterial strategies is urgent.

Against this backdrop, bacteriophage (phage) therapy has re-emerged as a promising antibiotic alternative, harnessing viruses that selectively infect and kill bacteria and drawing on a long history of clinical use in Europe and the former Soviet Union^5^. Yet, despite this legacy, phage therapy was never widely adopted by Western medicine, largely due to the convenience and broad-spectrum efficacy of antibiotics^5^. Today, however, the paradigm is shifting: phage therapy as both standalone and adjunctive treatment is experiencing renewed interest worldwide. This is not just an historical curiosity, but is a clinically viable option for infections that standard-of-care antibiotics are unable to cure. This resurgence is being driven especially by compelling case studies that have successfully resolved infections deemed untreatable by conventional means^6–9^, offering hope to physicians facing increasingly limited therapeutic options.

Amid this growing optimism, phage therapy still faces critical but often underreported challenges. These include inconsistent clinical outcomes, regulatory hurdles, the limited commercial incentives for developing new antibacterial agents generally, and a scarcity of large-scale, randomized controlled clinical trials^5^. Regulatory challenges are particularly acute at the global level, as there is currently no harmonized international framework for the approval and clinical use of phage therapy products. The difficulty in applying conventional pharmacological parameters—such as pharmacokinetics (PK) and pharmacodynamics (PD) to phage products given the self-replicating nature of phages and the not infrequent need for patient-specific formulations—further complicates regulatory pathways^5^. To move forward, phage therapy therefore needs more than just current emphases on strong basic research and ad hoc translational efforts. Required also is a multidisciplinary framework that fully integrates clinical medicine not only with microbiology but also with immunology, genomics, regulatory science, and even phage-bacterial ecological dynamics. Phage therapy *can* become a frontline weapon in the global fight against bacterial infections, but only through transparent and broadly collaborative translational science.

Well-celebrated clinical phage therapy triumphs^6–9^, while inspiring, thus represent incomplete narratives. To advance the field meaningfully, focus must also turn to understanding where treatments fall short. These less-successful outcomes are not merely setbacks; rather, their systematic analysis can help refine therapeutic protocols and inform development of more pharmacologically predictive and mechanistically grounded preclinical models. Just as important is phage therapy’s dynamic and adaptive nature—bacterial and patient responses can shift during treatment, requiring real-time therapeutic adjustments, ones that mirror established practices in antibiotic stewardship where clinicians routinely cycle agents to achieve efficacy in complex cases^2^.

## Failure is a blueprint: learning from what does not work

**Fig. 1:**
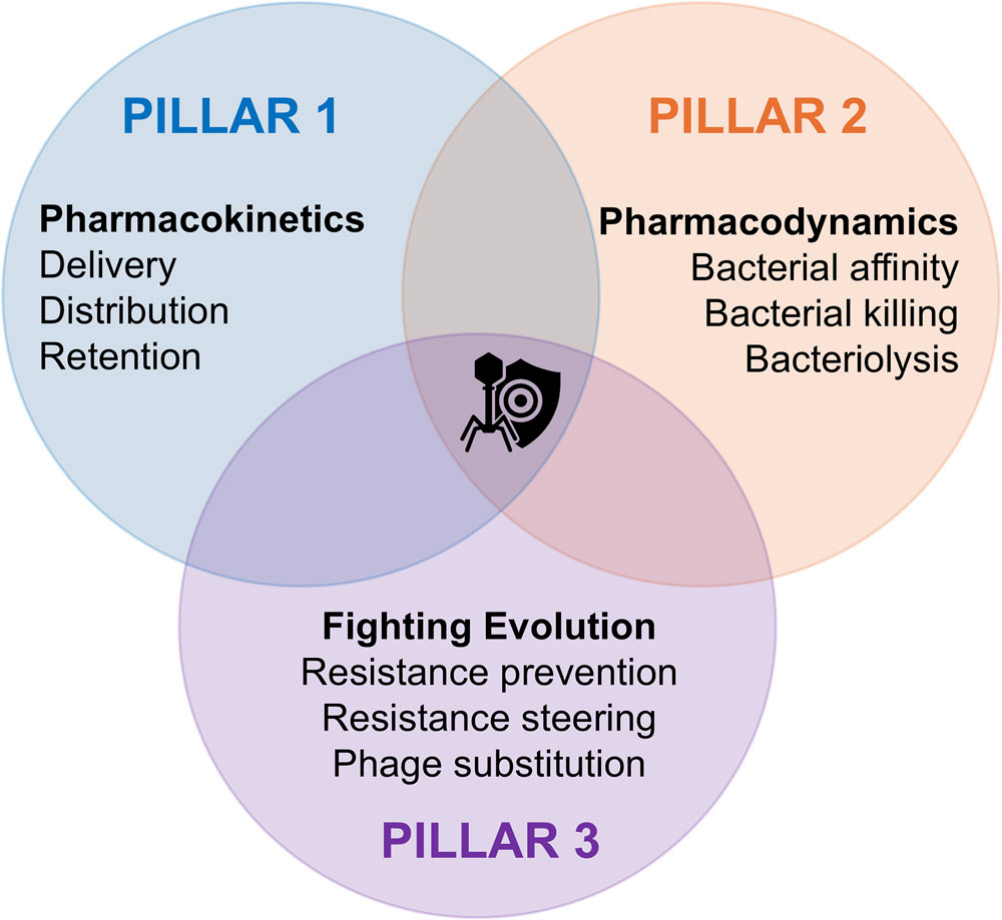
Three pillars of effective phage therapy. **1**. getting phages to bacteria, **2**. destroying bacteria once there, and **3**. interfering with bacterial evolution of resistance to phages.

The primary goal in phage therapy development should be achieving consistency in therapeutic outcomes. Yet, as clinical use remains far from routine, achieving such predictability will require better sharing of clinical insights, not only in terms of what works, but also in terms of what does not. Toward that goal of clinical dependability, we have identified recurring patterns that align with three foundational pillars of phage therapy success. These are: (Pillar 1) the familiar pharmacokinetics (PK; the study of how a therapeutic agent is absorbed, distributed, metabolized, and eliminated within the body) and (Pillar 2) pharmacodynamics (PD; (the study of a therapeutic agent’s effects on the target organism), but also (Pillar 3) fighting bacterial evolution of phage resistance (Fig. 1). Specifically, these issues include:

### Pillar 1. Dosing challenges

Achieving and sustaining therapeutic phage concentrations in situ, in the vicinity of targeted bacteria, is crucial to the success of any antibacterial agent. When phage concentrations are too low, bacterial burdens may be ineffectively reduced, undermining treatment outcomes. This issue in fact has limited efficacy in several trials, including the PhagoBurn study^10^, which demonstrated some effectiveness but failed to achieve superiority to standard antibiotics. Similarly, the randomized controlled trial by Leitner et al. (2021) of intravesical Pyophage for urinary tract infections failed to demonstrate superiority over placebo—variable titers across the cocktail’s component phages likely resulted in subtherapeutic concentrations to impact targeted pathogens^11^. Beyond the PhagoBurn and Pyophage trials, additional studies have highlighted the importance of dosing: for example, the controlled clinical trial by Wright et al. (2009) in chronic otitis reported dose-dependent effects^12^, while Petrovic Fabijan et al. (2020) demonstrated that intravenously administered phages were rapidly cleared from the bloodstream in patients with severe *Staphylococcus aureus* bacteraemia, suggesting that single-dose regimens may be insufficient to sustain therapeutic concentrations systemically^13^. The optimal dosing strategy or strategies necessary to consistently achieve sufficient phage delivery nonetheless remain uncertain, indicating a significant gap in our understanding of in vivo phage *pharmacokinetics*^12–15^.

### Pillar 2. Matching phage properties to clinical contexts

Even if phages reach bacteria in sufficient numbers, therapeutic success depends on precise pathogen matching—with phages much more so than antibiotics. This is a pharmacodynamic concern and is especially an issue for empirical treatments employing fixed formulations of phages. The randomized study by Sarker et al. (2016)^16^ in Bangladesh, for example, illustrated this challenge. Patients had diarrheal illnesses that turned out to not be caused by the targeted phage-susceptible *Escherichia coli*, eliminating the potential for meaningful phage impact. In immunocompromised individuals^3^, even well-targeted phages can require administration over months^17,18^, or may not work at all^19^, further highlighting the need for robust strategies to match phages to the specific challenges of infection (i.e., personalized therapy). This complexity is compounded by the fact that phage susceptibility can be highly context-dependent, shifting with bacterial physiology, such as due to biofilm formation^20^ or intracellular persistence^21^ as compared to exponentially growing bacteria in nutrient-rich laboratory medium. Thus, even phages accessing targeted bacteria in sufficient number (a pharmacokinetic concern) can be insufficient to achieve phage therapy success (a therefore pharmacodynamic concern). Moving forward, precision in pathogen identification, and resulting phage selection, are essential requirements toward optimizing phage therapy *pharmacodynamic* capabilities.

### Pillar 3. Staying ahead of resistance

Phage treatments, like antibiotic therapies, can be negatively impacted by evolutionary dynamics. Bacteria can rapidly adapt, resulting in resistance to treatment phages emerging mid-treatment. Indeed, the emergence of phage resistance during treatment has been documented in several clinical settings. For example, Aslam et al. (2024) reported that phage resistance contributed to treatment failure in five *Pseudomonas aeruginosa* ventricular assist device infections^22^, while resistance evolution during treatment has also been observed in *Mycobacterium abscessus* and *Klebsiella pneumoniae* cases^7,23,24^. These examples underscore the need for resistance-aware treatment design from the outset. To address this, phage therapies need to be designed to forestall the occurrence of phage resistance; thus, *Fighting Evolution*.

### Choosing the right phage or phages

Phage selection can represent a multifaceted and indeed ongoing challenge, one that ideally takes into account all three foundational pillars, PK, PD, and fighting the bacterial potential for resistance evolution. The principal challenge to phage therapy success therefore is one of consistently identifying the right phage or phages to treat disparate and often multi-drug resistant bacterial infections. These are phages that can reach bacteria in sufficient numbers (Pillar 1), phages that can effectively cull those bacteria once there (Pillar 2), and phages that at the same time can resist being thwarted by subsequent evolution of phage resistance (Pillar 3). Initiatives like the Global Clinical Phage Therapy Rounds^25^ are a promising step toward achieving this goal of more robust and consistent therapeutic outcomes, particularly against intransigent multidrug resistant bacterial infections, and this is achieved by facilitating exchange of real-world clinical experience among practitioners.

**Fig. 2:**
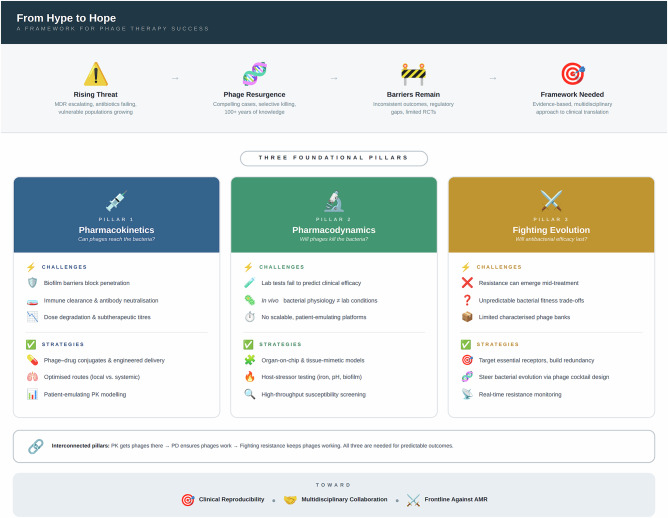
A framework for evidence-based phage therapy. Three foundational pillars—pharmacokinetics, pharmacodynamics, and fighting resistance evolution—must be addressed through interdisciplinary collaboration to achieve predictable, reproducible clinical outcomes against antibiotic-resistant bacterial infections. RCT randomized controlled trials, MDR multidrug-resistant, PK pharmacokinetics, PD pharmacodynamics.

As phage therapy moves closer to clinical integration, it will be essential also to build capacity not only among clinicians but also among hospital pharmacists, who are likely to play a key role in phage formulation, dosing, and stewardship^26^. This further highlights the need for preclinical researchers to gain detailed access to clinical realities—including both successful and unsuccessful phage treatments—to produce more predictive and translational research. We suggest that emphasis of these efforts should be on the three foundational pillars of phage therapy success, as elaborated upon in Fig. 2.

## Toward achieving more consistent phage therapy success

PK, PD, and curtailing resistance evolution are general themes in all antimicrobial therapies, including for phages. Addressing these three pillars toward consistently achieving therapeutic success, however, varies with specific agents. For phages in phage therapy we can identify a number of potentially useful approaches, many of which require greater clinical and pre-clinical development to reach their translational potential.

### Pillar 1. Getting and keeping phages where they are needed

To address “Dosing challenges”—particularly toward overcoming pharmacokinetic obstacles—strategies must focus on understanding how therapeutic phages behave within the body. This is toward reaching targeted bacteria in sufficient numbers and then their persisting long enough to eradicate infections^5,15,27–29^. The diversity of phages, bodies, and infections, however, complicate generalizations and highlight the need for strategies that more effectively tailor phage selection and dosing to specific circumstances. That is, and equivalently just as for antibiotics, substantial phage antibacterial activity is of only limited use unless phages can be sustained in sufficient numbers in the vicinity of targeted bacteria.

Delivery challenges can be particularly acute in biofilm-associated infections. Biofilms create dense, protective matrices that act as biological (sorptive), physical (diffusive), and chemical (hydrolytic) barriers to phage penetration^27^. These obstacles are highly relevant in salvage treatments, where prior antibiotic treatments have failed, and in chronic or persistent infections^30^—such as prosthetic joint infections, diabetic foot ulcers, chronic rhinosinusitis, and non-tuberculous mycobacterial lung disease. In cystic fibrosis, for instance, thick mucus and bacterial extracellular polymers can physically block phage particles from reaching bacteria^27^. Yet, many of these same infections nonetheless can still respond to phage therapies, and particularly so when phages are selected that display enhanced biofilm penetration. Examples are *Pseudomonas aeruginosa* phage OMKO1 and *Staphylococcus aureus* phage Sb-1, which show enhanced biofilm penetration relative to antibiotics^31,32^. Phage choice consequently is likely critical when treating biofilm-associated infections, though that possibility has been poorly explored clinically^22^.

Declines in phage concentrations within the body—the other major pharmacokinetic concern—can result from dilution of phages, their physical compartmentalization, their inactivation, or their removal both before and after phages have reached their bacterial targets. Losses can result from stomach-acid inactivation (for oral delivery), inefficient absorption, or systemic clearance via the mononuclear phagocyte system. Adaptive immunological factors, particularly phage-specific antibodies, can also play key though inconsistent roles^15,27^. Importantly, each phage type as well as delivery method can be affected differently by these obstacles. Compensating for these losses in any case requires some combination of both dosing with sufficient phage numbers and dosing sufficiently often, just as is the case for antibiotics; but also phages, uniquely among antibacterial agents, can compensate for losses by increasing their numbers in the course of infecting and killing targeted bacteria^33^. But what numbers or degree of compensation are necessary? These issues remain poorly appreciated, particularly clinically.

While animal models can offer useful pharmacokinetic and anti-phage immunological information, their translational value nonetheless remains limited. Especially, they often fail to replicate the complexity of human infections, particularly chronic, biofilm-associated, or immunologically nuanced conditions. These limitations are even greater for current in vitro models, which tend to not even attempt to mimic many of the barriers to reaching and then persisting at infection sites. Addressing these challenges requires patient-focused frameworks along with clinical feedback to improve pharmacokinetic models as well as exploration of innovative delivery strategies. For example, phage-drug conjugates—also known as phage nanobots—represent an innovative delivery strategy, in which antibacterial agents are chemically tethered to phage particles via stimuli-responsive linkers. These linkers are designed to release their drug payloads in response to conditions at the infection site (such as pH or enzymatic activity), thereby improving both drug penetration and persistence in complex environments^34^.

### Pillar 2. Separating phage wheat from bacteriophage chaff

To bridge the gap between laboratory phage testing and clinical pharmacodynamic realities—thus better “Matching phage properties to clinical contexts”—there is a need for more clinically representative testing platforms^27,33^. In vitro assays, and even preclinical animal models, thus often fail to replicate the hostile and heterogeneous environments encountered during actual bacterial infections—particularly infections that are biofilm-associated. These limitations are not unique to phages, however, as they parallel the issue of antibiotic tolerance^35^, where standard susceptibility assays overlook bacterial survival strategies under stress. Predictive phage therapy nonetheless can be even more complex given the high biological complexity of phages and their tendencies to interact with bacteria in dynamic, context-dependent ways. That difference, however, still favorably contrasts with the typical xenobiotic nature of candidate drugs, where negative pharmacologically emergent properties, i.e., unanticipated, excessive toxicities upon introduction into patients, is often a substantial concern—but is not similarly much of a concern with naturally occurring therapeutic phages as they consist of just proteins and DNA^5^. Thus, with phages, in vitro and in vivo testing primarily focusses on efficacy—as is our emphasis here—whereas with antibiotics during both clinical use and development, toxicity concerns are generally of equivalent importance to efficacy, as well as the more difficult to quantify negative impacts of antibiotics on the human microbiome^28^.

Even at their best at representing the phage treatment of human infections, animal models still are impractical for routine, clinical use. Indeed, though many treatment phages have been evaluated in animal models, the vast majority of phages remain untested in such systems. These limitations are compounded when there is a need to match phages to individual patient isolates for personalized use. The vast number of unique phages potentially available for therapeutic use, compared to the increasing dearth of new antibiotics, thus underscores the urgent need for scalable, high-throughput, in vitro platforms that can reliably predict therapeutic efficacy under clinically relevant conditions. Specifically, the challenge with antibiotics tends to be that we do not have enough varieties to non-toxically overcome evolved antibiotic resistance, whereas the challenge with phages is that we don’t have enough resources to test all potentially useful phages in vivo prior to their clinical use.

Though offering scalability, there exist multiple limitations to applying traditional antibacterial testing to phage testing, such as with minimum inhibitory concentration determinations^36^, starting with the phage ability to increase their concentrations over time and thereby their phage-killing activity during such assays by replicating^37^. Nutrient-rich media and exponentially growing bacterial cultures also can fail to reflect phage-relevant physiological aspects of real infections. In vivo, that is, bacteria often exist in nutrient-limited and slow-growing states, or as the noted biofilms. Their physiologies are shaped as well by host stressors, such as acidification, oxidative, and nitrosative stress, antimicrobial peptides, and iron deprivation^38^. Together, these pressures drive bacterial physiological adaptation, altering bacterial growth dynamics and gene expression in ways that can significantly affect phage susceptibility^39,40^. Genetic screens also have shown that many bacterial genes expressed in situ are dispensable under laboratory conditions^41^, highlighting the potential for mismatch between in vitro testing environments and the biological context in vivo. As a result, phages that seem to perform well in vitro may fail clinically. Inappropriate screening environments alternatively can lead to exclusion of promising therapeutic candidates^37^.

To bridge these gaps, researchers are increasingly turning to more clinically relevant bacterial states, such as stationary-phase or biofilm-associated populations, as well as tissue-mimetic systems for at least preliminary efficacy testing. These ideally can better reflect, in vitro, the characteristics of bacterial infections in patients^38^, though it is important to keep in mind that with phages even the simplest of systems can be surprisingly complex and even misleading^42,43^. Developing rapid, high-throughput in vitro screening methods thus remains technically challenging. However, recent advances, such as microfluidic organ-on-chip platforms, organoid-based infection models, and automated phage susceptibility assays make even more complex in vitro modeling increasingly feasible^41,44,45^. Iron-depleted media and folate-deficient conditions, already used to simulate physiological constraints in antibiotic testing, could also be adapted for phage screening^46^. These approaches should enable rapid testing of phage-antibiotic interactions, which is essential for identifying synergistic as well as antagonistic efficacy effects—the latter an especially overlooked aspect of therapy design^47,48^. More advanced systems now incorporate immune cells and mechanical forces, promising unprecedented fidelity in mimicking infection environments within a quasi-in vitro-in vivo context^49^.

Without development of such tools for routine phage testing, we risk relying on suboptimal phages for treatment efficacy as well as missing more optimal phages which happen to fail superficial criteria, such as by producing only relatively small plaques but still displaying rapid lytic clearance in broth^37^. In cases where phages show diminished infectivity in such models, strategies of phage training or genetic engineering can be employed to either enhance their infectivity or, in some cases, broaden their host range^50^.

To effectively identify, prioritize, and further develop phages with the highest therapeutic potential, the field thus urgently needs robust and standardized testing platforms. While initiatives like the EUCAST Ad hoc Subcommittee on Phage Susceptibility Testing^51^ are working toward standardization, current methods remain limited in scalability, speed, or realism, thereby often failing to deliver results that are actionable in clinical settings. Establishing scientifically rigorous, high-throughput systems for pharmacodynamic phage testing must be a top priority if we are to effectively separate desirable phage wheat from ineffective bacteriophage chaff.

### Pillar 3. Making treatments resistance proof

Rather than treating bacterial resistance to phages as an inevitable complication, effective phage therapy demands proactive strategies to anticipate, minimize, and exploit its evolution, that is, by “Staying ahead of resistance”^52^. If not addressed before treatments begin, then resistance can complicate monitoring, necessitate protocol adjustments, prolong treatment durations, and lead to less than desirable outcomes. Given the diversity of treatment targets and the complexities of phage-bacterial interactions, predicting the impact of resistance evolution on treatment outcomes represents a yet additional complication on phage choice. While several anti-resistance strategies exist^42,52^, a lack of systematic clinical exploration has left their impact on patient outcomes largely unknown.

Notwithstanding those concerns, the default approach to addressing evolution of bacterial resistance is simply to hope that it is not going to be a problem. This hope is not purely idealistic, however. For instance, even only partial bacterial clearance may allow immune systems to eliminate remaining, resistant bacteria. That “strategy”, though, is less effective in immunocompromised patients and also has too often failed, in the case of antibiotic treatments, to completely clear bacterial infections. Bacterial surface modifications that block phage infections – the most common form of bacterial resistance to phages – nonetheless have been documented in many cases to reduce bacterial virulence or restore bacterial sensitivity to antibiotics, so-called phage steering of resistance outcomes^5,50^. Such hopeful resistance results nevertheless are not guaranteed across all phage-bacteria-patient combinations. More than hope, therefore, is required for consistent phage therapy success in light of the bacterial potential to evolve phage resistance.

To move beyond simply hope for favorable resistance outcomes, two complementary approaches can be employed—one well-established and one more theoretical^40^. The first, phage substitution^52^, involves replacing phages once resistance emerges, just as physicians routinely introduce new antibiotics when confronted with intransigent cases against which current antibiotic treatments appear to have failed. Phage substitution indeed is standard practice in settings where phage therapy is routine, so long as alternative phages are readily available. However, broader implementation of this strategy is often hindered by limited availability of well-characterized and purified phage collections, a limitation which otherwise represents a main barrier to expanding phage clinical use generally. In addition, as phage-resistant bacteria are expected to almost always be present within bacterial populations of sufficient size, often phage-resistance does not present as a problem until it actually becomes a problem. Phage substitution thus tends to be a reactive approach to bacterial evolution of phage resistance.

The second approach is to proactively minimize resistance evolution^52^. This can involve uses of phages targeting bacterial receptors that serve essential functions for the bacterium, such that mutations conferring resistance impose significant bacterial virulence costs. Incorporating receptor redundancy within cocktails—selecting phages that bind distinct receptor types—can further constrain bacterial resistance evolution. The objective there is to reduce the potential for bacteria to successfully mutate to phage resistance, as is a goal as well for antibiotic combination treatments, such as when treating tuberculosis. Thus, these represent proactive selective or mutational constraints of bacterial evolution of phage resistance. Despite their promise, these strategies have seen only limited clinical translation.

An anti-resistance approach that has seen substantial clinical use is the combining of phages not just with other phages but also with antibiotics. This too is an anti-evolution strategy as bacteria usually are unable to pleiotropically evolve mechanisms to evade both agents. Furthermore, numerous studies have demonstrated that bacterial evolution of phage resistance can restore sensitivity to antibiotics—a sufficiently predictable trade-off that phage–antibiotic combinations can be deliberately designed to exploit it, effectively steering bacterial evolutionary trajectories toward more treatable phenotypes^42^. Overall, however, proactive anti-resistance strategies of any type have yet to be meaningfully integrated into clinical phage therapy. For a comprehensive overview of strategies to combat phage resistance evolution, including examples of resistance trade-offs and their clinical exploitation, we refer readers to recent dedicated reviews^52,53^.

In clinical settings, real-time monitoring also is critical for guiding phage therapy strategies in response to evolving resistance. This is especially relevant in personalized treatments, where phage selection and dosing ideally will align with specific infection contexts. Such monitoring, however, must extend beyond identification of mere presence of phage-resistant bacteria—which are expected to be nearly always present within bacterial populations at low levels—to include some means of identifying frequencies, absolute numbers, and even, as noted, whether resistance-mutant presence is clinically problematic. This difficult monitoring challenge points to a further utility of better proactively addressing phage resistance, such as through the use of phage cocktails^54^, rather than relying on strategies of phage substitution.

## Conclusion: building a resilient future for phage therapy

Phage therapy stands at a pivotal moment, poised between promise and proof. As antibiotic resistance continues to rise and populations continue to age, resulting in increased prevalences of infections that resist standard antibiotic treatments, the ongoing need for alternative antimicrobial strategies has never been more urgent. Yet, for phage therapy to transition from a niche intervention to a mainstream medical tool, we must confront and resolve the biological challenges that currently limit its predictability, scalability, and clinical reliability. This is particularly toward augmenting antibiotic use or addressing multi-drug resistance, including especially as salvage therapies.

Here we have outlined three foundational pillars that demand focused attention: better understanding of phage pharmacokinetic barriers (Pillar 1), development of pharmacodynamically relevant preclinical models of realistic bacterial physiologies (Pillar 2), and both the anticipation and management of bacterial resistance to phages (Pillar 3). These are not isolated technical hurdles; they are deeply interconnected challenges that shape the therapeutic potential of phages at every stage, from bench to bedside.

Timely addressing these issues requires more than just competitive but somewhat isolated scientific innovation. It demands instead a biomedical culture, across disciplines, of transparency, interdisciplinary collaboration, and shared learning between researchers and clinicians. For phage therapy to become more widespread, physicians nonetheless should not be required to become phage biologists—just as physicians should not be required to become pharmacologists to prescribe antibiotics—but hospital pharmacists, as well as clinical microbiology laboratories, will need targeted support as key players in ensuring safe and effective phage treatments. Just as clinical case discussions have begun to shape real-world phage therapy through initiatives like the Global Phage Clinical Rounds, the research community must establish parallel platforms to critically examine the biological mechanisms behind therapeutic outcomes. Only through this dual lens—clinical and scientific—can we refine phage therapy into a robust, adaptable, and globally accepted treatment modality.

Ultimately, phage therapy brings to antibacterial medicine the combined utilities of high mechanistic antibacterial diversity and low inherent toxicity to the body, both as have been understood with increasing appreciation now for over 100 years. The future of phage therapy thus is less dependent on new phage discoveries, but instead on how the phage and medical communities can come to work together to apply our collective and ideally still-improving knowledge of how best to apply phages to cure what otherwise have been incurable bacterial infections. By investing in improving the biological foundations of successful phage therapy today, we can build a more resilient, responsive, and effective therapeutic landscape for tomorrow.

### Related Links

**World Health Organization Regional Office for Europe. Building the evidence for the use of bacteriophage therapy**: WHO/EURO:2025-11441-51213-78039 (2025). https://www.who.int/europe/publications/i/item/WHO-EURO-2025-11441-51213-78039. **The antimicrobial potential of bacteriophages report: UK government response:** https://www.gov.uk/government/publications/the-antimicrobial-potential-of-bacteriophages-report-government-response.
